# Supplementary material for: Dynamic unidirectional anisotropy in cubic FeGe with antisymmetric spin-spin-coupling
Source: Sci Rep. 2020 Feb 18;10:2861. doi: 10.1038/s41598-020-59208-8 (PMC7029033; doi:10.1038/s41598-020-59208-8)
Supplement: Supplementary file 1 — Supplementary information. [file 41598_2020_59208_MOESM1_ESM.pdf]

Supplementary information: Dynamic unidirectional anisotropy in cubic FeGe with antisymmetric spin-spin-coupling

Nicolas Josten[1], Thomas Feggeler[1], Ralf Meckenstock[1], Detlef Spoddig[1], Marina Spasova[1], Ke Chai[2], Iliya Radulov[3], Zi-An Li[2], Oliver Gutfleisch[3], Michael Farle[1], and Benjamin Zingsem[1,4,*]

[1]Faculty of Physics and Center for Nanointegration (CENIDE), University Duisburg Essen, Duisburg, 47057, Germany
[2]Institute of Physics, Chinese Academy of Sciences, Beijing 100190, China
[3]Department of Material- and Geosciences, Functional Materials, Technische Universität Darmstadt
[4]Ernst Ruska Centre for Microscopy and Spectroscopy with Electrons and Peter Grünberg Institute, Forschungszentrum Jülich GmbH, 52425 Jülich, Germany
[*]Benjamin.Zingsem@uni-due.de

S1 Calculation of a distribution of resonance lines

The magnetocrystalline anisotropy of FeGe takes the form of eq. S1[1], where $K_4(T)$ and $K_6(T)$ are the temperature dependent anisotropy constants and $\vec{e}(\theta,\varphi) = (\sin(\theta)\cos(\varphi), \sin(\theta)\sin(\varphi), \cos(\theta))$ the unit vector of spherical coordinates.

$$\begin{aligned} F_{\text{cubic}} =& K_4(T)\cdot\left(e_{\text{x}}(\theta,\varphi)^2 e_{\text{y}}(\theta,\varphi)^2 + e_{\text{x}}(\theta,\varphi)^2 e_{\text{z}}(\theta,\varphi)^2 + e_{\text{y}}(\theta,\varphi)^2 e_{\text{z}}(\theta,\varphi)^2\right) \\ &+ K_6(T)\cdot\left(e_{\text{x}}(\theta,\varphi)^2 e_{\text{y}}(\theta,\varphi)^2 e_{\text{z}}(\theta,\varphi)^2\right) \end{aligned} \tag{S1}$$

One can now determine the resonance field by solving the LLG[2–4] using eq. S1 with an additional Zeeman-term for an FMR like excitation[5]. This has to be done for a statistically representative amount of crystallographic directions to determine the relative frequency of resonance fields in a polycrystalline material. For a spherical sample this would be sufficient. But in a bulk sample it must be accounted for the contribution of a demagnetization field. Assuming that during measurement the external magnetic field points along the easy direction of the shape anisotropy, in this case a flat cylinder (see fig. 2 b) inset), two different approaches can be used to calculate the resonance field distribution. The first approach is to rotate the magnetocrystalline part of the free energy density, so that the respective crystalline direction investigated points along both the hard axis and the external magnetic field. The second method is vice versa the rotation of both the hard axis and the external magnetic field to point along the respective crystalline direction. The latter was preferred. The corresponding free energy is then given by equation S2. $\vec{B}$ is the external field, $\underline{\underline{N}}$ the demagnetization tensor and $\underline{\underline{R_N}}$ and $\underline{\underline{R_B}}$ are rotation matrices rotating the demagnetization tensor and the external field in a way that they always align with the respective crystallographic direction.

$$F_{\text{ext}} = -\frac{\mu_0}{2}\cdot\vec{M}\cdot\underline{\underline{R_N}}\cdot\underline{\underline{N}}\cdot\underline{\underline{R_N}}^{\text{T}}\cdot\vec{M} - \vec{M}\cdot\underline{\underline{R_B}}\cdot\vec{B}(0,0) + F_{\text{cubic}} \tag{S2}$$

The distribution of resonance lines obtained for 10^5 sample points can be seen in fig. S1 a). The known cubic anisotropy constants of FeGe[1] of $K_4(276\,\text{K}) = -200\,\text{J}\,\text{m}^{-3}$ and $K_6(276\,\text{K}) = 2000\,\text{J}\,\text{m}^{-3}$ were used together with the demagnetization tensor N ($N_{zz} = 0.676, N_{xx,yy} = 0.162$) related to our sample. The distribution of resonance fields was then convoluted with the usual Dyson[6,7] lineshape to calculate the FMR spectra expected during measurement. Figure S1 a) shows a comparison between these calculated resonance spectra and the measured resonance spectra in the easy direction of shape anisotropy.

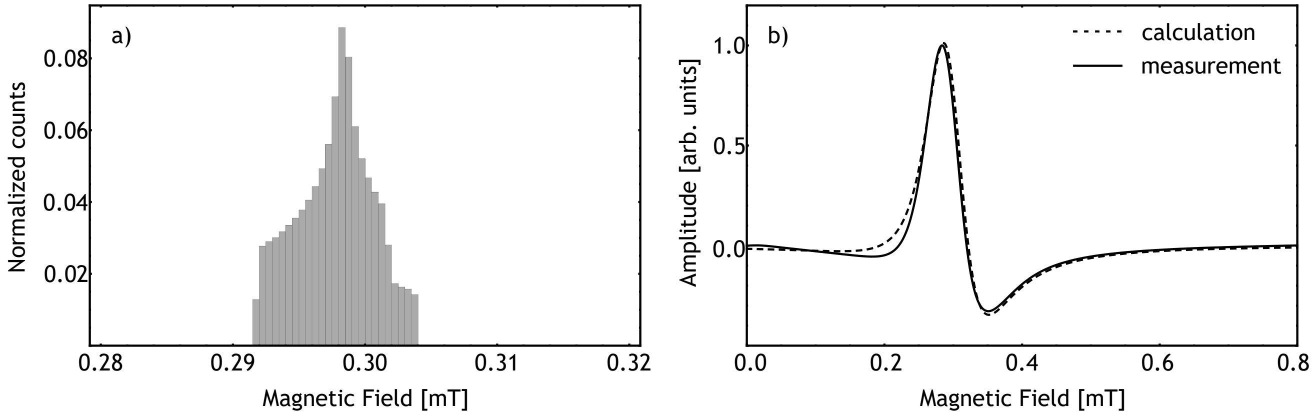

Figure S1. a) A hystogram of the frequency of occurrence of certain resonance fields of a statistically representative amount of crystallographic directions. b) ferromagnetic resonance spectra taken from fig. 2 a) at 90°. It is compared with the resonance spectra obtained using a Dysonian lineshape convoluted with the distribution of resonance lines in b).

S2 Unidirectional anisotropy

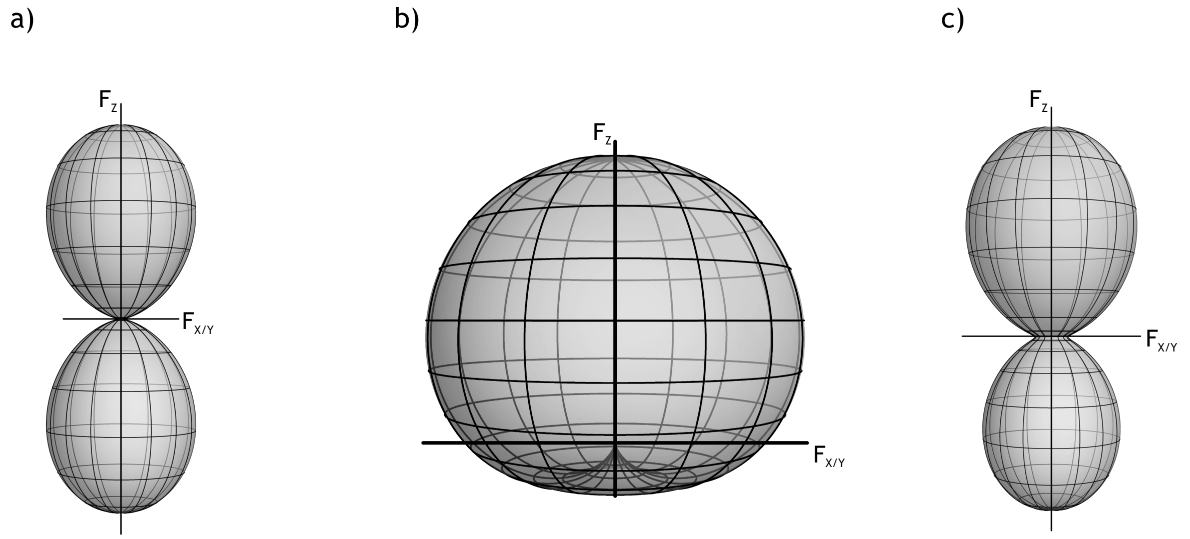

Figure S2. Schematic illustration of different anisotropic contributions to the free energy density landscape: a) Uniaxial anisotropy (proportional to $\cos^2(\theta)$), the dominating contribution in our measurement, which is represented as the demagnetization term in our model. The angle $\theta = 0°$ coincides with the z-direction. b) The proposed dynamic unidirectional anisotropy, which is proportional to $\cos(\theta)$. c) The combination of uniaxial and unidirectional anisotropy for $K_U = 10K_{UD}$.

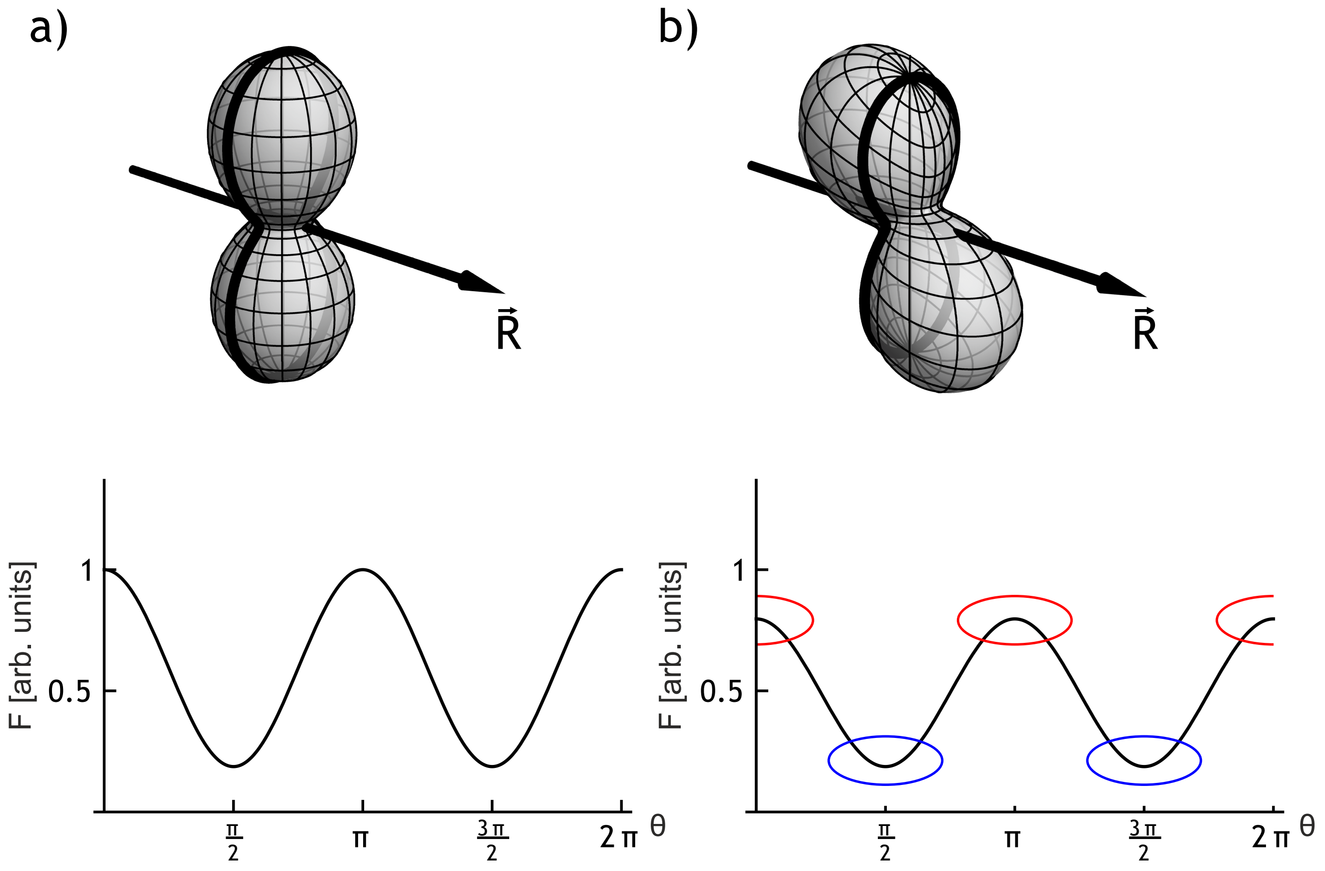

Figure S3. Schematic illustration of the inversion symmetry of the free energy density of a cylinder without unidirectional anisotropy a) with the rotation axis $\vec{R}$ parallel to the base of the cylinder and b) tilted from this position. In both cases the angular dependent spectrum follows the same symmetry. Note that a tilt of the magnetic field along an additional axis is therefore not equivalent to the observed unidirectional shift of the resonance line position reported in the main text.

References

1. Ludgren, L., Beckman, O., Attia, V., Bhattacheriee, S. P. & Richardson, M. Helical spin arrangement in cubic FeGe. *Phys. Scripta* **1**, 69–72, DOI: https://doi.org/10.1088/0031-8949/1/1/012 (1970).
2. Smit, J. & Beljers, H. G. Ferromagnetic resonance absorption in BaFe12O19, a highly anisotropic crystal. *Philips Res. Reports* **10**, 113–130 (1955).
3. Landau, L. & Lifshitz, E. Anomalous high-frequency resistance of ferromagnetic metals. *Phys. Z. Sowjetunion* **8**, 153–169 (1935).
4. Gilbert, T. L. A phenomenological theory of damping in ferromagnetic materials. *IEEE Transactions on Magn.* **40**, 3443–3449, DOI: https://doi.org/10.1109/TMAG.2004.836740 (2004).
5. Zingsem, B. W., Winklhofer, M., Meckenstock, R. & Farle, M. Unified description of collective magnetic excitations. *Phys. Rev. B* **96**, 224407, DOI: https://doi.org/10.1103/PhysRevB.96.224407 (2017).
6. Dyson, F. J. electron spin resonance absorption in metals. II. theory of electron diffusion and the skin effect. *Phys. Rev.* **98**, 349–359, DOI: https://doi.org/10.1103/PhysRev.98.349 (1955).
7. Joshi, J. P. & Bhat, S. On the analysis of broad dysonian electron paramagnetic resonance spectra. *J. Magn. Reson.* **168**, 284 – 287, DOI: https://doi.org/10.1016/j.jmr.2004.03.018 (2004).
